# Efficacy of different frequencies of extracorporeal shockwave on plantar flexor spasticity of the ankle in patients with stroke: a single-center, prospective, single-blind, randomized controlled trial

**DOI:** 10.1186/s12984-026-01901-2

**Published:** 2026-03-30

**Authors:** Meihua Ke, Peike Zhou, Dongxia Li, Weiji Kong, Congbai Xie, Yulong Wang, Zhongbo Wang, Jianjun Long

**Affiliations:** 1 Department of Rehabilitation, Pengzhou People’s Hospital, Pengzhou, Chengdu,, China; 2 Department of Pediatrics, Pengzhou Maternal and Child Health Care Hospital, Pengzhou, Chengdu,, China; 3 Department of Rehabilitation, Shenzhen Second People’s Hospital, Shenzhen, China; 4 Department of Rehabilitation, GaoMing Hospital of Traditional Chinese Medicine, Foshan, China; 5 Department of Rehabilitation, Dehua County Hospital, Quanzhou, China

**Keywords:** Ankle plantar flexors, Extracorporeal shock wave therapy, Spasticity, Stroke rehabilitation

## Abstract

**Background:**

Extracorporeal shockwave therapy (ESWT) can effectively relieve post-stroke spasticity; however, research comparing the impact of different ESWT frequencies is limited. In this study, we investigated the therapeutic efficacy of different ESWT frequencies for post-stroke ankle plantar flexor spasticity.

**Methods:**

In this prospective, single-blind, randomized controlled trial, we recruited patients treated in the Rehabilitation Department of Shenzhen Second People’s Hospital for post-stroke ankle plantar flexor spasticity between January 2024 and September 2024. The participants were randomly assigned to the control (*n* = 15), 4 Hz ESWT (*n* = 15), and 10 Hz ESWT (*n* = 15) groups. All groups received conventional rehabilitation therapy; the 4 Hz and 10 Hz ESWT groups received additional ESWT. The ESWT intervention lasted for 3 weeks, with two interventions per week. ESWT was administered at 2 bar, 4 Hz, and 2000 pulses for the 4 Hz group and 2 bar, 10 Hz, and 2000 pulses for the 10 Hz group. Assessments were conducted before treatment, after the first treatment, and at the end of intervention using the modified Ashworth scale (MAS), passive joint range of motion (PROM), ankle clonus score, medial gastrocnemius muscle oscillation frequency (MGF), lateral gastrocnemius muscle oscillation frequency (LGF), medial gastrocnemius muscle dynamic stiffness (MGS), lateral gastrocnemius muscle dynamic stiffness (LGS), and the Fugl-Meyer assessment of the lower extremity (FMA-LE). Visual analog scale (VAS) tool was used before and after each ESWT session.

**Results:**

The 4 Hz and 10 Hz ESWT groups showed significant difference in LGS after a single treatment (*P* < 0.05). Following 3 weeks of treatment, both ESWT groups exhibited significant improvements in MAS, PROM, MGF, and LGS compared with the control group (*P* < 0.05). The 4 Hz ESWT group also demonstrated a significant improvement in MGS compared with the control group (*P* < 0.05), and the 4 Hz and 10 Hz ESWT groups differed significantly in LGS (*P* < 0.05). We found that only five patients reported mild pain, which resolved rapidly after treatment and rest.

**Conclusion:**

Both single and 3-week ESWT treatments safely reduced the severity of ankle plantar flexor spasticity. The results showed the possibility of superiority of 4 Hz intervention to 10 Hz intervention.

*Trial registration:* This study was registered in the Chinese Clinical Trial Registry on 10 January 2024 (registration Number: ChiCTR2400079721), https://www.chictr.org.cn/bin/project/edit?pid=217074.

**Supplementary Information:**

The online version contains supplementary material available at 10.1186/s12984-026-01901-2.

## Background

Globally, stroke is a significant cause of mortality and disability [1], with muscle spasticity representing a common stroke complication that affects approximately 6.8–64% of patients following the onset of stroke [2]. Spasticity is typically associated with damage to upper motor neurons, which disrupts the balance between excitatory and inhibitory signals transmitted from the brain to spinal cord centers. This imbalance increases motor neuron excitability, leading to elevated muscle tone and hyperreflexia [3]. In patients with ankle plantar flexor spasticity, the foot contacts the ground in an inwardly rotated position, shortening the stance phase on the affected limb. This contributes to the development of a hemiplegic gait pattern, frequently characterized by a tendency to “walk in circles” [4]. Reducing spasticity in the plantar flexor muscles could improve ankle dorsiflexion range of motion and correct abnormal pronated foot strike patterns. These improvements will directly translate into a longer stance phase, enhanced gait symmetry and greater walking efficiency. Ultimately, these improvements can help patients to enhance their functional capacity, such as independent walking distance and speed, and their participation levels, such as reintegration into the community [5]. Therefore, managing spasticity in patients with stroke is crucial.

Extracorporeal shockwave therapy (ESWT) can effectively alleviate post-stroke muscle spasticity [6, 7]. Few investigations have investigated its application in this condition, exploring the type of ESWT, optimal treatment sites, duration of therapeutic effects, and key parameters influencing treatment outcomes [8–11]. Based on the wave transmission mechanism, ESWT is classified into two distinct modalities: ​​focused ESWT (fESWT)​​ and ​​radial ESWT (rESWT)​​. Although fESWT delivers higher energy to a targeted area, rESWT offers broader but more superficial penetration [8], making it a preferable minimally invasive option for conservative treatment [12]. Both fESWT and rESWT effectively reduce post-stroke muscle spasticity [13]. However, a meta-analysis suggests that rESWT holds greater therapeutic potential for this condition [7], potentially yielding superior clinical outcomes compared with fESWT [8]. In studies focusing on spastic equinovarus deformity following stroke, both fESWT and rESWT significantly improved modified Ashworth scale (MAS) and Modified Tardieu Scale scores. Notably, rESWT demonstrated more therapeutic advantages in improving passive range of motion (PROM) of the ankle joint and optimizing foot pressure distribution during walking [14]. ESWT has also been demonstrated to exhibit significant tissue-targeted differences in efficacy. One study had demonstrated that a dual-target intervention strategy, combining the muscle-tendon junction and middle of the muscle belly, can optimize treatment outcomes [9]. ​​Meta-analyses have shown that ESWT has sustained efficacy in the management of post-stroke spasticity, including short-term (within 2 weeks), medium-term (2–4 weeks), and long-term (4 weeks to 3 months) assessments [10]. ESWT has also been demonstrated to rapidly improve spasticity symptoms in patients with stroke, exhibiting a significant immediate effect [15, 16]. Regarding treatment parameters, Yang et al. [17] reported that 4000 pulses ESWT was more effective than 2000 pulses ESWT in improving the function of patients with post-stroke ankle spasticity.

Determining optimal treatment protocols remains a critical research area in the application of ESWT for stroke rehabilitation [18]. Among the adjustable parameters of ESWT (such as intensity and pulse), frequency is one of the core physical parameters determining the depth at which the acoustic wave’s mechanical force acts upon tissue [19]. This significance has been corroborated in other fields: previous studies have confirmed that the frequency parameters of ESWT have a significant impact on the clinical efficacy for patients with plantar fasciitis [20] and pediatric urinary tract stones [21]. These findings lead us to hypothesize that the frequency of ESWT may also influence the efficacy of treating post-stroke spasticity. However, the specific impact of ESWT frequency in this area has not been sufficiently explored. Consequently, we investigated the effects of extracorporeal shock waves at different frequencies on treating ankle plantar flexor spasticity in patients with stroke.

Various treatment protocols using rESWT have employed frequencies ranging from 4 Hz to 10 Hz [15]. Both 4 Hz and 10 Hz ESWT are safe, effective, practical, and non-invasive for reducing post-stroke spasticity [6, 7]. However, empirical evidence comparing the efficacy of these different frequencies is scarce. Therefore, in this study, we selected treatment protocols with the highest frequency disparity to investigate the immediate and medium-term effects of ESWT at different frequencies on plantar flexor spasticity in patients with stroke.

## Methods

### Design

This single-center, prospective, single-blind, randomized controlled trial employed simple randomization using *IBM SPSS Statistics for Windows*,* version* 29.0 (IBM Corp., Armonk, NY, USA), with allocation concealment ensured through the use of opaque envelopes. The sample size calculation for this study employed G*Power 3.1 software (University of Düsseldorf, Düsseldorf, Germany), based on prior statistical analyses of ESWT treatment for lower limb spasticity in patients with stroke [9]. The effect size was set at f = 0.72, with α = 0.05 and test power *P* = 0.80, across three groups. Accounting for potential patient attrition during the study, with an anticipated dropout rate of 20%, the total sample size for the entire study was determined to be 45 cases. A research assistant (not involved in assessments or interventions) generated a 1:1:1 randomization sequence using simple randomization to assign 45 participants into three groups: control (*n* = 15), 4 Hz ESWT (*n* = 15), and 10 Hz ESWT (*n* = 15). Randomization cards were sealed and stored by an independent assistant (unaware of group assignments) who disclosed the allocations after participant enrollment and informed consent. Result assessment was conducted independently by a blinded assessor, while statistical analysts were granted non-blinded access to data during the data processing phase.

All procedures followed the ethical principles outlined in the 2013 Declaration of Helsinki. The study proceeded with the enrolment process after all participants or their legal guardians had provided written informed consent, thereby ensuring that participants voluntarily participate based on full knowledge and understanding of the study objectives and potential risks. All treatment and assessment therapists have obtained at least a primary therapist certification.

### Participants

**Fig. 1:**
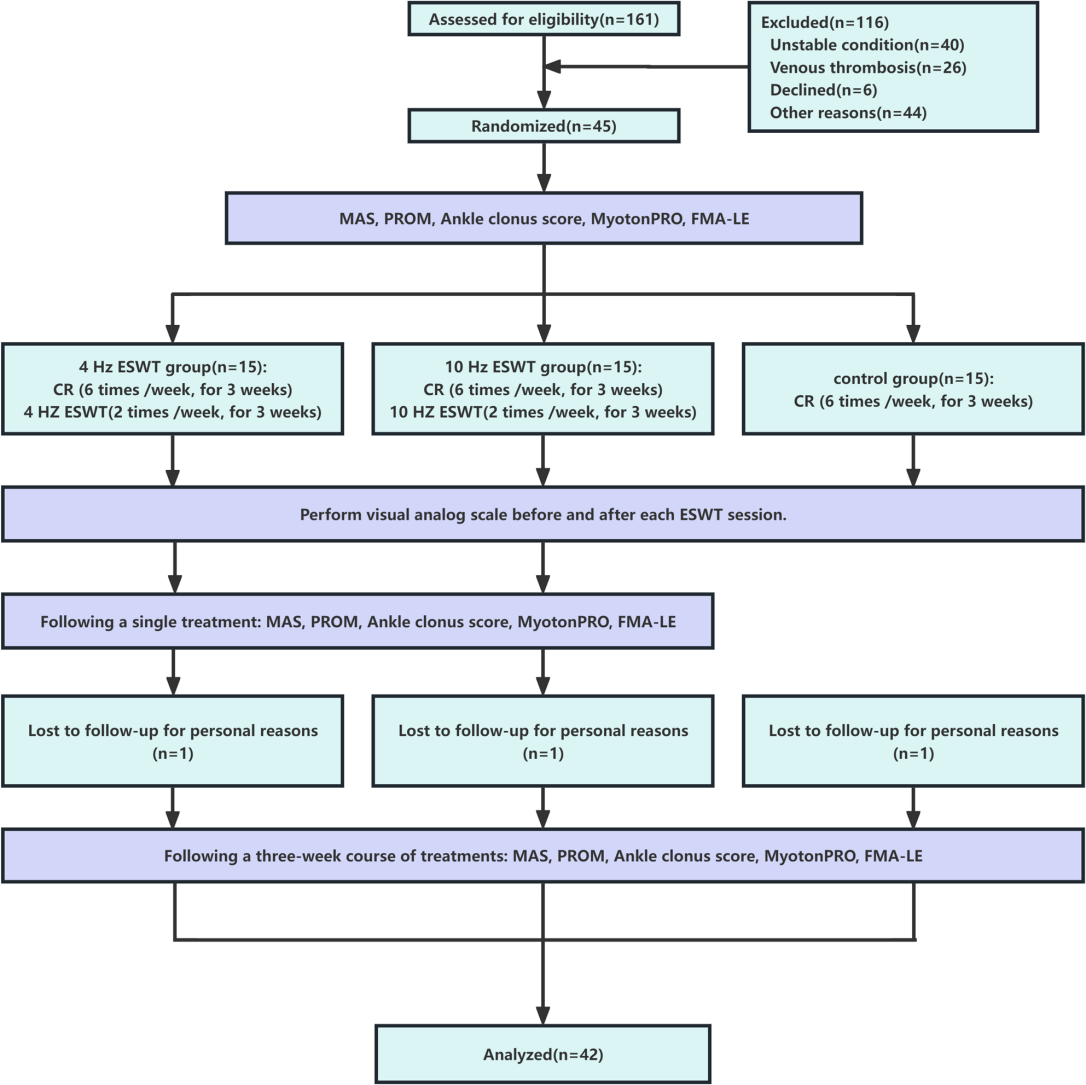
Flow diagram.* MAS* modified Ashworth scale,* PROM* passive range of motion,* FMA-LE* Fugl-Meyer assessment for lower extremity,* ESWT* extracorporeal shock wave therapy,* CR* conventional rehabilitation

We recruited 45 patients treated in the Rehabilitation Department of Shenzhen Second People’s Hospital for post-stroke ankle plantar flexor spasticity between January 2024 and September 2024. The inclusion criteria were as follows: (1) first-time stroke accompanied by hemiparesis, (2) ankle plantar flexor spasticity graded Ⅰ–Ⅲ according to MAS, (3) stable vital parameters, (4) aged 30–75 years, (5) disease duration ranging 2–48 weeks, (6) adequate cognitive ability to follow simple verbal instructions (Mini-Mental State Examination score ≥ 24), and (7) agreement to sign the informed consent. The exclusion criteria were as follows: (1) dyskinesia or conditions directly influencing motor function; (2) history of epilepsy; (3) malignant tumors; (4) presence of a cardiac pacemaker; (5) coagulation disorders; (6) lower limb fracture or venous thrombosis; (7) ankle contracture; (8) inflammation or infection in the treatment area; and (9) prior use of oral antispasmodic drugs, botulinum toxin injections, local nerve blocks, or anti-spasticity surgery. Detachment criteria were the following: (1) cases where treatment was not administered according to the trial protocol after enrollment; (2) cases where poor subject compliance, voluntary withdrawal, or failure to complete the entire treatment course compromised efficacy or safety assessment; and (3) cases where informed consent was withdrawn during the study. Discontinuation criteria were the following: (1) sudden deterioration of the patient’s condition; (2) occurrence of serious adverse reactions; and (3) cases where the patient requests to discontinue participation in the study. Figure 1 presents a flow diagram illustrating the study methodology.

### Interventions

All groups received conventional rehabilitation (CR), which was individualized based on patient needs and included physical therapy, occupational therapy, speech training, and acupuncture. CR involved a 3-week intervention, 6 days per week, with 4 h of therapy per day. In addition to CR, the 4 Hz and 10 Hz ESWT groups received ESWT treatment, which comprised a 3-week course, with two sessions per week (one session per day and at least 2 days between sessions), totaling six therapy sessions.

**Fig. 2:**
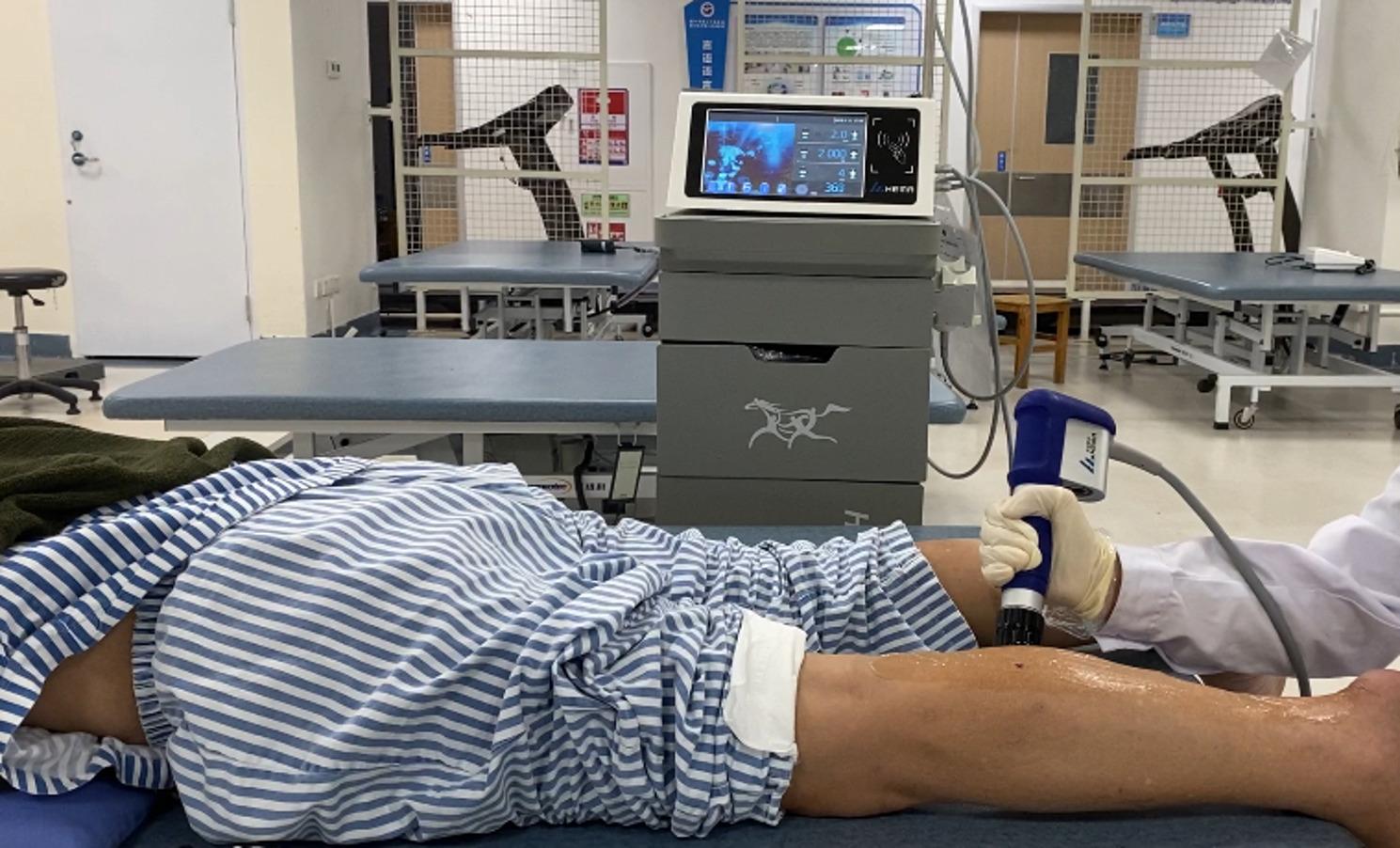
Schematic diagram of extracorporeal shockwave operation

The two ESWT groups received rESWT using the rESWT device, *HMJYS-A* (Zhuhai Hema Medical Instrument Co., Ltd., Zhuhai, China). Patients were positioned prone, and therapist precisely positioned the shock waves probe at the muscle–tendon junction and the middle area between the medial and lateral heads of the gastrocnemius muscle for intervention. The shock wave parameters were set at 2000 pulses, 2 bar, and 4 Hz for the 4 Hz ESWT group and 2000 pulses, 2 bar, and 10 Hz for the 10 Hz ESWT group [22]. (Fig. 2)

### Outcome measures

We used the MAS as the primary efficacy evaluation indicator, whereas the secondary outcome measures included ankle PROM, ankle clonus score, MyotonPRO, and Fugl-Meyer assessment of the lower extremity (FMA-LE). All outcome measures were assessed at three time points: baseline (T0), subsequent to the first therapeutic application (T1), and at the end of the treatment cycle (T2). The VAS (visual analog scale) was administered before and after each ESWT session.

#### Primary outcome

The MAS demonstrates a reliable clinical tool for quantifying post-stroke spasticity severity [23]. During the assessment, participants were positioned supine with the knee extended. The assessor then moved the ankle from maximum plantarflexion to maximum possible dorsiflexion [15, 24]. The MAS was assessed on a 6-point rating scale (spasticity levels of 0, Ⅰ, Ⅰ^+^, Ⅱ, Ⅲ, and Ⅳ); these ratings were recorded as 0, 1, 2, 3, 4, and 5, respectively, for the statistical analysis.

#### Secondary outcomes

##### PROM

Ankle PROM was assessed using the *Right Gait & Posture* system (Shenzhen Xingzheng Technology Co., Ltd., Shenzhen, China). Participants were positioned supine, with the sensor placed vertically beneath the affected foot. Measurements began with the ankle in a neutral position, followed by passive dorsiflexion and plantarflexion while maintaining the left–right axis stability. PROM was calculated as the sum of the maximal dorsiflexion and plantarflexion angles recorded by the sensor [25].

##### Ankle clonus score

The ankle clonus score assesses severity by counting the number of sustained twitching movements [26]. Clonus duration was rated on a 5-point scale: 0, no ankle clonus; 1, clonus lasting 1–4 s; 2, 5–9 s; 3, 10–14 s; and 4, ≥ 15 s [27].

##### MyotonPRO

The *MyotonPRO* (Myoton Co., Ltd., Tallinn, Estonia) is a non-invasive device designed to measure certain characteristics of muscle tissue. As an objective quantitative tool, it can assess muscle tension and stiffness [28]. It is a compact, portable apparatus with high sensitivity, stable measurement capabilities, and proven reliability [29, 30]. During the measurement process, the probe should be positioned vertically above the measurement point on the muscle. The device subsequently generates a brief, mild pulse to stimulate the muscle being tested. Due to its elastic properties, the muscle undergoes damped vibration in conjunction with the probe at this point. The built-in accelerometer in the device captures and records the muscle’s mechanical vibration patterns, which are then analyzed and calculated by the software. Common assessment metrics include oscillation frequency and dynamic stiffness. Oscillation frequency refers to the natural frequency at which muscle tissue undergoes damped oscillations following mechanical pulsation. It reflects muscle tension levels, with higher values indicating greater tension [31]. In this study, it was measured for the medial gastrocnemius (MG) and lateral gastrocnemius (LG) muscles, denoted as MG muscle oscillation frequency (MGF)​ and LG muscle oscillation frequency (LGF), respectively. Dynamic stiffness denotes the muscle’s resistance to deformation under an external force; higher values signify greater stiffness [32]. Muscle dynamic stiffness exhibits a close positive correlation with muscle tension, thus dynamic stiffness also reflects the level of muscle tension [33]. The dynamic stiffness of the MG and LG muscles is denoted as MG muscle dynamic stiffness (MGS)​ and LG muscle dynamic stiffness (LGS), respectively.

During the measurement process, patients were positioned prone, with the feet extending beyond the edge of the bed [34]. The measurement point for the MG was set at the 70% mark of the entire length of the calf (from the lateral malleolus to the popliteal fossa) [35]. The measurement point for the LG was also located at the 70% mark of the calf’s length, as measured from the Achilles tendon to the fibular head [36]. Two test points were marked, and the probe was placed vertically over each mark. The average of the three tests at each measurement point was taken as the final result. MGF, LGF, MGS, and LGS were recorded.

##### FMA-LE

The FMA-LE is a composite score that assesses the reflex activity, joint activity, coordination, and speed in the lower extremities. The total score ranged from 0 to 34, with higher scores indicating superior motor function [37].

#### Safety evaluation indicator

We assessed adverse events following each ESWT session. Through direct questioning and physical examination of the treatment site, we actively monitored known therapy-related adverse events (including local pain, subcutaneous ecchymosis, and swelling). We used the VAS tool for pain assessment.

### Statistical analysis

Statistical analyses were conducted using *IBM SPSS Statistics for Windows*,* version* 29.0 (IBM Corp., Armonk, NY, USA) . Normality distribution was assessed via the Shapiro–Wilk test. Continuous variables are expressed as means (standard deviations) or medians (interquartile ranges); unordered categorical variables are presented as frequency (percentages); while ordered categorical variables are described using medians (interquartile ranges) or frequency (percentages). Variance homogeneity was assessed by Levene’s test.

To compare baseline characteristics among the three patient groups, one-way ANOVA, Kruskal-Wallis H test, Pearson’s chi-square test, or Fisher’s exact test applied for between-group differences, as appropriate. Paired-samples *t* tests or Wilcoxon signed-rank tests were used for within-group comparisons of two groups, whereas independent samples *t* tests or Mann–Whitney U tests were employed for between-group analyses. For three-group comparisons, the same paired/non-parametric approach was used for within-group assessments. When comparing the efficacy of the three treatment groups after 3 weeks, the Kruskal–Wallis H test was used to assess overall differences among the groups, as the data represented ordered categorical variables or continuous variables that did not follow a normal distribution or had unequal variances. If the test results indicated statistical differences, Dunn’s test was further applied for pairwise comparisons between groups. To control for Type I error inflation due to multiple comparisons, the significance level for Dunn’s test was adjusted using the Bonferroni correction. Significance was set at *P* < 0.05. When comparing immediate effects and 3-week effects, *P* < 0.05 was still used for within-group comparisons, while Bonferroni correction was uniformly applied for between-group comparisons, adjusting the significance level to α = 0.025. For the intergroup analysis of 3-week efficacy, we first performed the Kruskal–Wallis H test (judgment threshold: *P* < 0.025). If statistically significant differences were detected, pairwise comparisons were conducted, and the adjusted p-values were compared with 0.05 for judgment.

In addition to reporting statistical significance, effect sizes were calculated to quantify the observed effect strength. Effect sizes were interpreted using widely adopted conventions: η²F and *ε²*values of 0.01, 0.06, and 0.14 represented small, medium, and large effects, respectively; *V* (df = 2) thresholds were 0.07, 0.21, and 0.35; Cohen’s *d* thresholds were 0.20, 0.50, and 0.80; and *r* thresholds were 0.10, 0.30, and 0.50 [38]. Analysis was strictly conducted according to the groups initially randomized. All analyses were performed on the available data only, and participants with missing outcome data were excluded from the respective analyses.

### Important changes to the trial after it commenced

During data collection, we found that the pre-specified secondary outcome measure (the Long Scale Activities of Daily Living Score) lacked sufficient sensitivity within the 3-week intervention period. Consequently, we discontinued collection of this outcome measure and shifted focus to the FMA-LE, which is more sensitive to changes in lower limb function. Additionally, deviations from inclusion criteria occurred during recruitment. To accurately reflect the study cohort and enhance clinical relevance, the pre-registered criterion of “disease duration ≤ 12 months” was adjusted to the actual enrollment range of “2–8 weeks” during manuscript preparation.

## Results

### Comparison of participants’ general characteristics and baseline values

We ultimately included 45 participants; one patient from each of the three groups was lost to follow-up due to personal reasons (discharge or transfer to another hospital), and the remaining 42 (33 men and 9 women) completed all scheduled assessments and intervention sessions (Fig. 1). The mean ages of the participants were 56.21 (9.33), 47.36 (9.92), and 50.00 (10.58) years in the control, 4 Hz ESWT, and 10 Hz ESWT groups, respectively. Most patients had a MAS of Grade Ⅰ, distributed as follows: 9 (64.3%) in the control group, 7 (50.0%) in the 4 Hz ESWT group, and 3 (21.4%) in the 10 Hz ESWT group. Baseline characteristics including age, sex, stroke type, stroke side, stroke duration, or clinical measures (MAS, PROM, ankle clonus score, MyotonPRO, and FMA-LE) showed no intergroup differences across the three groups (*P* > 0.05; Table 1).

**Table 1:** Characteristics of the patients at baseline

	Control group (*n* = 14)		4 Hz ESWT group (*n* = 14)		10 Hz ESWT group (*n* = 14)		Statistical tests		*P*		Effect sizes		
Age, mean (SD), years	56.21 (9.33)		47.36 (9.92)		50.00 (10.58)		F = 2.919		0.066		η²_F_ = 0.130^ΔΔ^		
Sex, no. (%)							Fisher’s exact test (2 × 3 table)		0.334		*V* = 0.246^ΔΔ^		
Male	10 (71.4)		13 (92.9)		10 (71.4)								
Female	4 (28.6)		1 (7.1)		4 (28.6)								
Type of stroke, no. (%)							*χ²* = 0.571​		0.751		*V* = 0.117^Δ^		
Infraction	6 (42.9)		8 (57.1)		7 (50.0)								
Hemorrhage	8 (57.1)		6 (42.9)		7 (50.0)								
Side of stroke, no. (%)							*χ²* = 1.336		0.513		*V* = 0.178^Δ^		
Right	9 (64.3)		6 (42.9)		7 (50.0)								
Left	5 (35.7)		8 (57.1)		7 (50.0)								
Duration, median (IQR), days	69.50 (122.75)		56.50 (83.50)		82.00 (185.00)		*H* = 0.103		0.950		*ε²* ≈ 0.000^Δ^		
MAS, no. (%)							*H* = 4.673		0.097		*ε²* ≈ 0.069^ΔΔ^		
Ⅰ	9 (64.3)		7 (50.0)		3 (21.4)								
Ⅰ^+^	2 (14.3)		2 (14.3)		4 (28.5)								
Ⅱ	3 (21.4)		4 (28.5)		6 (42.9)								
Ⅲ	0 (0.0)		1 (7.1)		1 (7.1)								
PROM, mean (SD)	61.29 (7.62)		58.36 (8.05)		57.71 (13.81)		*F* = 0.485		0.619		η²_F_ = 0.024^Δ^		
Ankle clonus score, no. (%)							*H* = 5.857		0.053		*ε²* ≈ 0.099^ΔΔ^		
0	4 (28.6)		7 (50.0)		2 (14.3)								
1	4 (28.6)		4 (28.6)		3 (21.4)								
2	4 (28.6)		0 (0.0)		2 (14.3)								
3	2 (14.3)		0 (0.0)		1 (7.1)								
4	0 (0.0)		3 (21.4)		6 (42.9)								
MyotonPRO													
MGF, Hz, mean (SD) or median (IQR)	15.04 (1.12)		17.00 (3.47)		15.26 (1.86)		*H* = 1.901		0.387		*ε²* ≈ 0.000^Δ^		
LGF, mean (SD), Hz	15.72 (1.24)		16.15 (2.00)		16.17 (2.00)		*F* = 0.229		0.755		η²_F_= 0.014^Δ^		
MGS, N/m mean (SD) or median (IQR)	248.17 (53.33)		288.83 (29.67)		267.91 (23.58)		*H* = 2.160		0.340		*ε²* ≈​​ 0.004^Δ^		
LGS, N/m mean (SD) or median (IQR)	295.60 (23.02)		314.17 (26.92)		298.41 (27.32)		*H* = 1.503		0.472		*ε²* ≈ 0.000^Δ^		
FMA-LE, mean (SD)	18.29 (7.73)		20.29 (7.29)		18.71 (4.75)		*F* = 0.344		0.711		η²_F_ = 0.017^Δ^		

*ESWT* extracorporeal shock wave therapy,* SD* standard deviation,* IQR* interquartile range,* MAS* modified Ashworth scale,* PROM* passive range of motion,* MGF* medial gastrocnemius muscle oscillation frequency,* LGF* lateral gastrocnemius muscle oscillation frequency,* MGS* medial gastrocnemius muscle dynamic stiffness,* LGS* lateral gastrocnemius muscle dynamic stiffness,* FMA-LE* Fugl-Meyer assessment for lower extremity. Continuous variables are expressed as means (SD) or medians (IQR); unordered and ordered categorical variables are presented as no. (%). *P* < 0.05 indicates significant difference between the three groups. ^Δ^ indicates a small effect size, ^ΔΔ^ indicates a medium effect size, and ^ΔΔΔ^ indicates a large effect size

### Comparison of immediate effect outcome indicators

After a single treatment with ESWT, both the 4 Hz (MAS, *Z* = – 2.000, *P* < 0.05, *r* = – 0.535, large effect) and 10 Hz (MAS, *Z* = – 2.336, *P* < 0.05, *r* = – 0.624, large effect) ESWT groups showed improvement in MAS, PROM, and MyotonPRO (MGF, LGF, MGS, and LGS) compared with those at baseline (*P* < 0.05), whereas both ankle clonus scores and FMA-LE showed no statistical variations (*P* > 0.05) (Table 2). Comparison between the 4 Hz and 10 Hz ESWT groups revealed a statistical difference in LGS (*t* = – 2.405, *P* < 0.025, Cohen’s *d* = – 0.909, large effect) indicating a large effect size, with no significant differences observed in other outcomes (*P* > 0.05) (Table 3). Following a single treatment session, most patients achieved a MAS of Grade Ⅰ, distributed as follows: 9 (64.3%) in the 4 Hz ESWT group and 3 (21.4%) in the 10 Hz ESWT group (Table 4).

**Table 2:** Within-group comparison of changes in efficacy after one treatment

	4 Hz ESWT group (*n* = 14)							10 Hz ESWT group (*n* = 14)						
	T0	T1	T1-T0	Statistical tests	*P*	Effect sizes		T0	T1	T1-T0	Statistical tests	*P*	Effect sizes	
MAS, median (IQR)	1.50 (2.00)	1.00 (1.20)	0.00 (1.00)	*Z* = – 2.000	0.046*	*r* = – 0.535^ΔΔΔ^		2.50 (1.25)	2.00 (0.50)	0.00 (1.00)	*Z* = – 2.336	0.025*	*r* = – 0.624^ΔΔΔ^	
PROM, mean (SD) or median (IQR)	58.36 (8.05)	63.29 (8.52)	4.93 (0.74)	*t* = 6.685	<0.001*	Cohen’s *d* = 1.787^ΔΔΔ^		57.71 (13.81)	69.00 (22.00)	6.43 (0.66)	*t* = 9.731	*<*0.001*	Cohen’s *d* = 2.601^ΔΔΔ^	
Ankle clonus score, median (IQR)	0.50 (1.75)	0.00 (1.75)	0.00 (0.00)	*Z* = – 1.000	0.317	*r* = – 0.267^Δ^		2.50 (3.00)	2.00 (3.00)	0.00 (0.25)	*Z* = – 1.732	0.083	*r* = – 0.463^ΔΔ^	
MyotonPRO														
MGF, Hz, mean (SD) or median (IQR)	17.00 (3.47)	14.25 (2.08)	– 0.83 (3.22)	*Z* = – 3.182	0.001*	*r* = – 0.850^ΔΔΔ^		15.26 (1.86)	14.51 (1.91)	– 0.75 (0.91)	*t* = – 3.078	0.009*	Cohen’s *d* = – 0.823^ΔΔΔ^	
LGF, Hz, mean (SD) or median (IQR)	16.15 (2.00)	16.19 (1.97)	– 0.59 (1.05)	*Z* = – 3.298	<0.001*	*r* = – 0.881^ΔΔΔ^		16.17 (2.00)	15.41 (1.95)	– 0.76 (0.91)	*t* = – 3.120	0.008*	Cohen’s *d* = – 0.834^ΔΔΔ^	
MGS, N/m, mean (SD) or median (IQR)	288.83 (29.67)	263.00 (56.92)	– 16.00 (31.43)	*Z* = – 3.297	<0.001*	*r* = – 0.881^ΔΔΔ^		267.91 (23.58)	254.43 (24.57)	– 13.48 (6.78)	*t* = – 7.442	<0.010*	Cohen’s *d* = – 1.989^ΔΔΔ^	
LGS, N/m, mean (SD) or median (IQR)	314.17 (26.92)	281.95 (28.46)	– 24.33 (12.27)	*t* = – 7.422	<0.001*	Cohen’s *d* = – 1.984^ΔΔΔ^		298.41 (27.32)	283.68 (28.29)	– 14.73 (8.53)	*t* = – 6.457	<0.010*	Cohen’s *d* = – 1.726^ΔΔΔ^	
FMA-LE, mean (SD)	20.29 (7.29)	20.29 (7.29)	0	–	–			18.71 (4.75)	18.71 (4.75)	0	–	–		

*ESWT* extracorporeal shock wave therapy,* MAS* modified Ashworth scale,* IQR* interquartile range,* PROM* passive range of motion,* SD* standard deviation,* MGF* medial gastrocnemius muscle oscillation frequency,* LGF* lateral gastrocnemius muscle oscillation frequency; MGS, medial gastrocnemius muscle dynamic stiffness, * LGS* lateral gastrocnemius muscle dynamic stiffness,* FMA-LE* Fugl-Meyer assessment for lower extremity.* T0* evaluation at baseline;* T1* evaluation after one treatment. Continuous variables are expressed as means (SD) or medians (IQR); while ordered categorical variables are described using medians (IQR). *P* < 0.05 indicates significant difference between evaluation at baseline and evaluation after one treatment. * significant difference between evaluation at baseline and evaluation after one treatment. ^Δ^ indicates a small effect size, ^ΔΔ^ indicates a medium effect size, and ^ΔΔΔ^ indicates a large effect size

**Table 3:** Between-group comparison of changes in efficacy after one treatment

	4 Hz ESWT group (*n* = 14) T1-T0	10 Hz ESWT group (*n* = 14) T1-T0	Statistical tests	*P*	Effect sizes
MAS, median (IQR)	0.00 (1.00)	0.00 (1.00)	U = 91.000	0.691	*r* = – 0.075^Δ^
PROM, mean (SD)	4.93 (0.74)	6.43 (0.66)	*t* = – 1.515	0.142	Cohen’s *d* = – 0.573^ΔΔ^
Ankle clonus score, median (IQR)	0.00 (0.00)	0.00 (0.25)	*U* = 84.000	0.289	*r* = – 0.201^Δ^
MyotonPRO					
MGF, mean (SD) or median (IQR), Hz	– 0.83 (3.22)	– 0.75 (0.91)	*U* = 74.500	0.280	*r* = – 0.204^Δ^
LGF, mean (SD) or median (IQR), Hz	– 0.59 (1.05)	– 0.76 (0.91)	*U* = 94.500	0.872	*r* = – 0.030^Δ^
MGS, mean (SD) or median (IQR), N/m	– 16.00 (31.43)	– 13.48 (6.78)	*U* = 63.000	0.108	*r* = – 0.304^ΔΔ^
LGS, mean (SD), N/m	– 24.33 (12.27)	– 14.73 (8.53)	*t* = – 2.405	0.024*	Cohen’s *d* = – 0.909^ΔΔΔ^
FMA-LE, median (IQR)	0	0	–	–	–

*ESWT*, extracorporeal shock wave therapy,* MAS* modified Ashworth scale,* IQR* interquartile range,* PROM* passive range of motion,* SD* standard deviation,* MGF* medial gastrocnemius muscle oscillation frequency,* LGF* lateral gastrocnemius muscle oscillation frequency,* MGS*, medial gastrocnemius muscle dynamic stiffness,* LGS* lateral gastrocnemius muscle dynamic stiffness,* FMA-LE* Fugl-Meyer assessment for lower extremity.* T0* evaluation at baseline;* T1* evaluation after one treatment. Continuous variables are expressed as means (SD) or medians (IQR); while ordered categorical variables are described using medians (IQR). *P* < 0.025 indicates significant difference between the two groups. *significant difference within groups. ^Δ^ indicates a small effect size, ^ΔΔ^ indicates a medium effect size, and ^ΔΔΔ^ indicates a large effect size

**Table 4:** Distribution of MAS and ankle clonus scores following a single treatment session

	4 Hz ESWT group (*n* = 14)	10 Hz ESWT group (*n* = 14)
MAS, no. (%)		
0	0 (0.0)	0 (0.0)
Ⅰ	9 (64.3)	3 (21.4)
Ⅰ^+^	2 (14.3)	8 (57.1)
Ⅱ	2 (14.3)	3 (21.4)
Ⅲ	1 (7.1)	0 (0.0)
Ankle clonus score, no. (%)		
0	8 (57.1)	2 (14.3)
1	3 (21.4)	4 (28.6)
2	0 (0.0)	2 (14.3)
3	0 (0.0)	1 (7.1)
4	3 (21.4)	5 (35.7)

* ESWT* extracorporeal shock wave therapy,* MAS* modified Ashworth scale. Ordered categorical variables are described using no. (%)

### Comparison of outcome indicators after 3 weeks of treatment

After 3 weeks of treatment, improvements were observed in MAS (*Z* = -2.887, *P* < 0.05, *r* =-0.772 , large effect), PROM, ankle clonus scores, MGF, LGF, MGS, LGS, and FMA-LE in 4 Hz ESWT group. Similarly, after 3 weeks of treatment, improvements were observed in MAS (*Z* = -2.714, *P* < 0.05, *r* =-0.725 , large effect), PROM, ankle clonus scores, MGF, LGF, MGS, LGS, and FMA-LE in 10 Hz ESWT group. In the control group, improvements from baseline were noted in PROM, LGF, and FMA-LE but not in MAS, ankle clonus scores, MGF, MGS, and LGS in the control group compared with baseline (Table 5).

**Table 5:** Within-group comparison of changes in efficacy after 3 weeks of treatment

	Control group (*n* = 14)				4 Hz ESWT group (*n* = 14)					10 Hz ESWT group (*n* = 14)				
	T2-T0	Statistical tests	*P*	Effect sizes	T2-T0		Statistical tests	*P*	Effect sizes	T2-T0		Statistical tests	*P*	Effect sizes
MAS, median (IQR)	0.00 (0.00)	*Z* = – 1.000	0.317	*r* = – 0.267 ^Δ^	– 1.00 (1.00)		*Z* = – 2.887	0.004*	*r* = – 0.772 ^ΔΔΔ^	– 1.00 (1.00)		*Z* = – 2.714	0.007*	*r* = – 0.725 ^ΔΔΔ^
PROM, mean (SD)	2.86 (2.18)	*t* = 4.907	< 0.001*	Cohen’s *d* = 2.021^ΔΔΔ^	9.50 (7.65)		*t* = 4.644	< 0.001*	Cohen’s *d* = 1.241^ΔΔΔ^	9.50 (5.60)		*t* = 6.349	< 0.001*	Cohen’s *d* = 1.697^ΔΔΔ^
Ankle clonus score, median (IQR)	0.00 (0.25)	*Z* = – 1.732	0.083	*r* = – 0.463 ^ΔΔ^	0.00 (1.00)		*Z* = – 2.070	0.038*	*r* = – 0.553 ^ΔΔΔ^	– 1.00 (1.00)		*Z* = – 2.828	0.005*	*r* = – 0.756 ^ΔΔΔ^
MyotonPRO														
MGF, Hz, mean (SD)	– 0.15 (1.26)	*t* = – 0.437	0.670	Cohen’s *d* = – 0.117 ^Δ^	– 1.37 (1.63)		*t* = – 3.133	0.008*	Cohen’s *d* = – 0.837^ΔΔΔ^	– 1.16 (0.88)		*t* = – 4.914	< 0.010*	Cohen’s *d* = – 1.313^ΔΔΔ^
LGF, Hz, mean (SD)	– 0.98 (0.92)	*t* = – 4.003	0.002*	Cohen’s *d* = – 0.917^ΔΔΔ^	– 1.91 (1.77)		*t* = – 4.059	0.001*	Cohen’s *d* = – 1.767^ΔΔΔ^	– 1.78 (1.36)		*t* = – 4.919	< 0.001*	Cohen’s *d* = – 1.357^ΔΔΔ^
MGS, N/m, mean (SD)	– 2.74 (5.45)	*t* = – 1.879	0.083	Cohen’s *d* = – 0.502^ΔΔ^	– 29.05 (20.39)		*t* = – 5.329	< 0.001*	Cohen’s *d* = – 1.424^ΔΔΔ^	– 10.52 (11.63)		*t* = – 3.385	0.005*	Cohen’s *d* = – 0.905^ΔΔΔ^
LGS, N/m, mean (SD) or median (IQR)	– 2.67 (3.91)	*Z* = – 1.540	0.123	*r* = – 0.412^ΔΔ^	– 37.22 (16.22)		*t* = – 8.585	< 0.001*	Cohen’s *d* = – 2.294^ΔΔΔ^	– 16.24 (11.13)		*t* = – 5.459	< 0.001*	Cohen’s *d* = – 1.459^ΔΔΔ^
FMA-LE, median (IQR)	1.00 (1.25)	*Z* = 2.992	0.003*	*r* = 0.800 ^ΔΔΔ^	1.50 (3.00)		*Z* = 3.109	0.002*	*r* = 0.831 ^ΔΔΔ^	1.00 (3.25)		*Z* = 2.701	0.007*	*r* = 0.722 ^ΔΔΔ^

*ESWT* extracorporeal shock wave therapy,* MAS* modified Ashworth scale,* IQR* interquartile range,* PROM* passive range of motion,* SD* standard deviation,* MGF* medial gastrocnemius muscle oscillation frequency,* LGF* lateral gastrocnemius muscle oscillation frequency,* MGS* medial gastrocnemius muscle dynamic stiffness,* LGS* lateral gastrocnemius muscle dynamic stiffness,* FMA– LE* Fugl– Meyer assessment for lower extremity.* T0* evaluation at baseline,* T2* evaluation after 3 weeks treatment. Continuous variables are expressed as means (SD) or medians (IQR); while ordered categorical variables are described using medians (IQR). *P* < 0.05 indicates significant difference between evaluation at baseline and evaluation after 3 weeks. *significant difference between evaluation at baseline and evaluation after 3 weeks

^Δ^ indicates a small effect size, ^ΔΔ^ indicates a medium effect size, and ^ΔΔΔ^ indicates a large effect size

The 4 Hz and 10 Hz ESWT groups exhibited statistical differences in terms of MAS, PROM, and LGS compared with the control group (*P* < 0.05). However, these differences were not observed in ankle clonus, LGF, MGF, or FMA-LE scores (Table 6). The 4 Hz ESWT group demonstrated statistically different MGS compared with the control group (*P* < 0.05), whereas the 10 Hz ESWT group showed no statistical difference (*P* > 0.05) (Table 6). Only LGS showed statistical differences between the 4 Hz and 10 Hz ESWT groups (*P*< 0.05) (H = 26.362, *P* < 0.025, *ε²* = 0.625, large effect) (Table 6). All other parameters (MAS, PROM, ankle clonus scores, MGF, LGF, MGS, and FMA-LE) remained comparable among the groups (*P* > 0.05) (Table 6). After 3 weeks of treatment, most patients achieved a MAS of Grade Ⅰ, distributed as follows: 9 (64.3%) in the control group, 8 (57.1%) in the 4 Hz ESWT group, and 7 (50.0%) in the 10 Hz ESWT group (Table 7).

**Table 6:** Between-group comparison of changes in efficacy after 3 weeks of treatment

	Control group (*n* = 14) T2-T0	4 Hz ESWT group (*n* = 14) T2-T0	10 Hz ESWT group (*n* = 14) T2-T0	H	*P*	ε²	① vs. ② Adj. *p*	① vs. ③ Adj. *p*	② vs. ③ Adj. *p*
MAS, median (IQR)	0.00 (0.00)	– 1.00 (1.00)	– 1.00 (1.00)	10.672	0.005*	0.222	0.008 ^#^	0.025^#^	> 0.999
PROM, mean (SD)	2.86 (2.18)	9.50 (7.65)	9.50 (5.60)	13.248	0.001*	0.288	0.010 ^#^	0.002^#^	> 0.999
Ankle clonus score, median (IQR)	0.00 (0.25)	0.00 (1.00)	– 1.00 (1.00)	3.284	0.194	0.033	–	–	–
MyotonPRO									
MGF, Hz, mean (SD)	– 0.15 (1.26)	– 1.37 (1.63)	– 1.16 (0.88)	9.212	0.010	0.185	–	–	–
LGF, Hz, mean (SD)	– 0.98 (0.92)	– 1.91 (1.77)	– 1.78 (1.36)	3.774	0.152	0.045	–	–	–
MGS, N/m, mean (SD)	– 2.74 (5.45)	– 29.05 (20.39)	– 10.52 (11.63)	14.749	<0.001*	0.327	<0.001^#^	0.237	0.113
LGS, N/m, mean (SD) or median (IQR)	– 2.67 (3.91)	– 37.22 (16.22)	– 16.24 (11.13)	26.362	<0.001*	0.625	<0.001^#^	0.019^#^	0.049^#^
FMA-LE, median (IQR)	1.00 (1.25)	1.50 (3.00)	1.00 (3.25)	1.455	0.483	0.000	–	–	–

*ESWT* extracorporeal shock wave therapy,* MAS* modified Ashworth scale,* IQR* interquartile range,* PROM* passive range of motion,* SD* standard deviation,* MGF* medial gastrocnemius muscle oscillation frequency,* LGF* lateral gastrocnemius muscle oscillation frequency;* MGS* medial gastrocnemius muscle dynamic stiffness,* LGS* lateral gastrocnemius muscle dynamic stiffness,* FMA-LE* Fugl-Meyer assessment for lower extremity.* T0* evaluation at baseline,* T2* evaluation after 3 weeks treatment. ① indicates the control group, ② indicates the 4 Hz ESWT group, ③ indicates the 10 Hz ESWT group. Adj. p: Significance values have been adjusted by the Bonferroni correction for multiple tests.​ Continuous variables are expressed as means (SD) or medians (IQR); while ordered categorical variables are described using medians (IQR). *P* < 0.025 indicates significant difference between three groups. Adj. *P* < 0.05 indicates significant difference between groups. *significant difference between three groups. ^#^ significant difference between two groups

**Table 7:** Distribution of MAS and ankle clonus scores following 3 weeks of treatment

	Control group (*n* = 14)	4 Hz ESWT group (*n* = 14)	10 Hz ESWT group (*n* = 14)
MAS, no. (%)			
0	0 (0.0)	2 (14.3)	0 (0.0)
Ⅰ	9 (64.3)	8 (57.1)	7 (50.0)
Ⅰ^+^	3 (21.4)	3 (21.4)	4 (28.6)
Ⅱ	2 (14.3)	1 (7.1)	3 (21.4)
Ⅲ	0 (0.0)	0 (0.0)	0 (0.0)
Ankle clonus score, no. (%)			
0	4 (28.6)	10 (71.4)	4 (28.6)
1	6 (42.9)	1 (7.1)	3 (21.4)
2	3 (21.4)	2 (14.3)	1 (7.1)
3	1 (7.1)	0 (0.0)	3 (21.4)
4	0 (0.0)	1 (7.1)	3 (21.4)

Abbreviations: ESWT, extracorporeal shock wave therapy; MAS, modified Ashworth scale. Ordered categorical variables are described using no. (%)

### Safety of ESWT treatment for muscle spasticity following stroke

To date, no cases of severe pain have been reported following ESWT treatment for post-stroke muscle spasticity, nor have any serious or persistent complications been observed. Only five patients reported mild pain, which resolved shortly after treatment rest. Pain scores for affected patients are detailed in Table 8.

**Table 8:** Pain rating record form

Number	First treatment			Second treatment			Third treatment			Fourth treatment		Fifth treatment		Sixth treatment	
	Before treatment	After treatment		Before treatment	After treatment		Before treatment	After treatment		Before treatment	After treatment	Before treatment	After treatment	Before treatment	After treatment
1	0	2		0	2		0	2		0	2	0	2	0	2
2	0	2		0	2		0	2		0	2	0	2	0	2
3	0	2		0	2		0	2		0	2	0	2	0	2
4	0	1		0	1		0	1		0	1	0	1	0	1
5	0	1		0	1		0	1		0	1	0	1	0	1

## Discussion

We investigated the effects of ESWT at different frequencies on plantar flexor spasticity in patients after stroke. Our findings showed that a single treatment significantly reduced ankle plantar flexor spasticity and improved ankle PROM in both the 4 Hz and 10 Hz ESWT groups, corroborating the majority of prior research outcomes [15, 39]. Radinmehr et al. [15] administered rESWT (2000 pulses, 0.34 mJ/mm²) to patients with ankle plantar flexor spasticity following stroke and found significant improvements in spasticity and active range of motion and PROM of the ankle joint compared with baseline. Similarly, Radinmehr et al. [39] found that after a single session of rESWT (2000 pulses, 0.34 mJ/mm²) targeting the plantar flexor muscles showed improvements in muscle structure of the gastrocnemius and soleus muscles, active and ROM and Modified MAS scores.

However, reports on the therapeutic efficacy of a single ESWT treatment on the ankle joint PROM among patients after stroke who exhibit plantar flexor spasticity remain contradictory. Lee et al. [25] performed ESWT on the gastrocnemius muscles in patients with chronic stroke, setting the shock wave parameters to 4 Hz, 2000 pulses, and 0.1 mJ/mm² intensity. Their findings indicated that a single treatment with fESWT improved some ultrasound-based parameters and MAS scores for spasticity but did not improve ankle PROM. Two possible factors may explain this limited efficacy. First, the enrolled patients had a long post-stroke duration, potentially influencing the therapeutic outcomes. Second, the intervention selectively targeted only the MG head while omitting the lateral head, which may have contributed to suboptimal treatment outcomes.

Following a 3-week ESWT intervention, we found that ESWT was more effective than CR in improving muscle tone and ankle PROM in patients after stroke. These findings corroborate those of previous studies, demonstrating the therapeutic benefits of repeated ESWT interventions for spasticity management in this population. Nada et al. [40] randomly divided patients with stroke and calf muscle spasticity into two groups: Group 1 received a 4-week rESWT intervention, with weekly sessions (1500 pulses at 4 Hz and an energy density of 0.10–0.3 mJ/mm²), whereas Group 2 received sham rESWT. They found statistically significant between-group differences in MAS, passive ankle dorsiflexion, 10-meter walk test, and H/M ratio at 1 and 2 months post-treatment, thereby highlighting ESWT as a non-invasive adjunctive modality for mitigating plantar flexor spasticity and equinovarus deformity in patients after stroke. Yang et al. [17] divided patients with plantar flexor spasticity following stroke into experimental and control groups, administering 4000 pulses/session to the experimental group and 2000 pulses/session to the control group. They employed fESWT at 4 Hz frequency and 0.10-0.134 mJ/mm² energy density. Both groups received four ESWT sessions during a 2-week treatment period. The experimental group showed statistically significant improvements in MAS, timed Up and Go (TUG) test, PROM, and Barthel index following intervention, whereas the control group exhibited significant gains only in the Barthel index. Intergroup comparisons also revealed superior improvements in strain elastography outcomes, Barthel index, and the TUG test in the experimental group. Combining ESWT with other rehabilitation therapies may further enhance its efficacy. Future research should focus on optimizing treatment parameters and combination regimens to promote motor function recovery and improve outcomes in patients with stroke.

Current research evidence highlights that multiple ESWT treatments have a beneficial effect on post-stroke muscle spasticity, potentially by inducing nitric oxide production, causing transient neurotransmission dysfunction, and regulating the excitability of α motor neurons [41–43]. Nevertheless, the therapeutic effects of multiple ESWT treatments on ankle clonus and lower limb function improvement remain controversial. Our 3-week study found no significant between-group differences in ankle clonus or lower limb function between the ESWT and control groups, contrasting with the positive findings of previous studies. Mihai et al. [44] applied ESWT (10 Hz, 2000 pulses, and 1 bar, with one treatment per week for 2 weeks) to the tendon insertion points of the gastrocnemius and soleus muscles to Group 1 (rESWT, visual feedback balance training, and CR), whereas Group 2 received sham rESWT, visual feedback training, and CR. In contrast to our findings, Mihai et al. [44] reported that ESWT significantly improved ankle clonus. Possible reasons for this discrepancy are their use of visual feedback balance training and focus on comparing the combined effects without setting up a placebo control group.

Our findings demonstrated a statistically significant difference in LGS between the 4 Hz and 10 Hz ESWT groups after a single session and 3 weeks of treatment. This study provides the first direct comparative evidence demonstrating that 4 Hz therapy may be superior to 10 Hz therapy in alleviating post-stroke ankle plantar flexor spasticity. This suggests that low-frequency extracorporeal shock wave therapy may offer more significant clinical benefits in alleviating post-stroke calf muscle spasticity. The underlying mechanism lies in the fact that lower-frequency shock waves possess greater tissue penetration capabilities, enabling them to exert a moderate relaxing effect on spastic muscles and alleviate excessive mechanical stress [45]. This finding aligns with existing literature: studies report that ESWT achieves optimal efficacy in improving post-stroke limb spasticity, pain, motor function, and passive range of motion under parameters below 8 Hz frequency and less than 2 bar pressure [13]. Therefore, our research suggests that clinically adjusting ESWT frequency parameters represents a promising direction for optimizing treatment strategies for post-stroke muscle spasticity. This evidence-based recommendation enables clinicians to refine existing protocols, thereby enhancing intervention efficacy while providing a clear pathway toward achieving truly personalized spasticity management plans.

This study presents some limitations that warrant acknowledgment. First, the selected ESWT frequencies only represent the upper (10 Hz) and lower (4 Hz) limits of the common frequency range. Therefore, whether intermediate frequencies between 4 Hz and 10 Hz could produce different therapeutic effects is unclear. The generalizability of the study results is somewhat limited due to the lack of exploration of intermediate frequencies, and we could not comprehensively assess the optimal effects of different frequencies on post-stroke calf muscle spasticity. Secondly, the brief duration of the intervention and the lack of follow-up are further limitations. Thirdly, the MAS is a subjective clinical tool, and its reliability and validity have been called into question. Finally, the patients included in this study were predominantly concentrated in the MAS of Grade I, with a limited number of cases classified as MAS of Grade III. Therefore, although 4 Hz ESWT demonstrated advantages in the overall population, it remains inconclusive whether this frequency yields significantly superior efficacy compared with 10 Hz ESWT for treating plantar flexor spasticity of the ankle in MAS Grade III patients. Due to sample size limitations, this study was unable to perform effective subgroup analyses. Future studies should incorporate more balanced and larger patient cohorts to enable in-depth analysis of subgroups with varying spasticity severity, thereby determining the optimal frequency-efficacy relationship.

## Conclusion

Both single and multiple ESWT treatments over 3 weeks reduced the severity of ankle plantar flexor spasticity. Furthermore, ESWT using a 4 Hz frequency may demonstrate greater efficacy in alleviating ankle plantar flexor spasticity, which is of great significance for optimizing ESWT treatment strategies. This study represents the first randomized controlled trial to evaluate the effects of different ESWT frequencies on post-stroke plantar flexor spasticity of the ankle, providing a theoretical basis for optimizing ESWT parameter settings in treating this condition. Another advantage of this study lies in its pioneering use of MyotonPRO to assess the effect of ESWT on muscle tone in post-stroke plantar flexor spasticity of the ankle, thereby enabling quantitative measurement of improvement outcomes. To further validate the robustness and clinical relevance of this study’s results, future studies should consider designing more treatment groups, conducting multi-group-controlled trials with longer-term follow-up, extending the duration of interventions, and optimizing frequency parameters more precisely.

## Supplementary Information

Below is the link to the electronic supplementary material.

Supplementary Material 1.

## Data Availability

No datasets were generated or analyzed during the current study. Due to restrictions in the informed consent process, which specified that participant data would only be shared with researchers under a controlled access mechanism for specific ethical and scientific purposes. Anonymized data from this study will be made available upon request to the corresponding author after formal publication, subject to reasonable ethical considerations.

## Abbreviations

ESWT: Extracorporeal shockwave therapy
fESWT: Focused extracorporeal shockwave therapy
rESWT: Radial extracorporeal shockwave therapy
MAS: Modified Ashworth scale
PROM: Passive range of motion
MG: Medial gastrocnemius
LG: Lateral gastrocnemius
MGF: Medial gastrocnemius muscle oscillation frequency
LGF: Lateral gastrocnemius muscle oscillation frequency
MGS: Medial gastrocnemius muscle dynamic stiffness
LGS: Lateral gastrocnemius muscle dynamic stiffness
FMA- LE: Fugl-Meyer assessment of the lower extremity
CR: Conventional rehabilitation
