# Supplementary material for: Efficacy of different frequencies of extracorporeal shockwave on plantar flexor spasticity of the ankle in patients with stroke: a single-center, prospective, single-blind, randomized controlled trial
Source: J Neuroeng Rehabil. 2026 Mar 30;23:154. doi: 10.1186/s12984-026-01901-2 (PMC13159371; doi:10.1186/s12984-026-01901-2)
Supplement: Supplementary file 1 — Supplementary Material 1. [file 12984_2026_1901_MOESM1_ESM.docx]

**Table 5 Baseline and after 3 weeks treatment measurements by group**

|  | Control group (n=14) | | | 4 Hz ESWT group (n=14) | | | 10 Hz ESWT group (n=14) | | |
| --- | --- | --- | --- | --- | --- | --- | --- | --- | --- |
|  | T0 | T2 | T0 | | T2 | T0 | | T2 |  |
| MAS, median (IQR) | 1.00 (1.25) | 1.00 (1.00) | 1.50 (2.00) | | 1.00 (1.00) | 2.50 (1.25) | | 1.50 (1.25) |  |
| PROM, mean (SD) | 61.29 (7.62) | 64.14 (6.74) | 58.36 (8.05) | | 67.86 (11.09) | 57.71 (13.81) | | 67.21 (11.43) |  |
| Ankle clonus score, median (IQR) | 1.00 (2.00) | 1.00 (2.00) | 0.50 (1.75) | | 0.00 (1.25) | 2.50 (3.00) | | 1.50 (3.25) |  |
| MyotonPRO |  |  |  | |  |  | |  |  |
| MGF, Hz, mean (SD) or median (IQR) | 15.04 (1.12) | 14.90 (1.73) | 17.00 (3.47) | | 14.60 (1.20) | 15.26 (1.86) | | 14.10 (1.80) |  |
| LGF, Hz, mean (SD) or median (IQR) | 15.72 (1.24) | 14.74 (1.31) | 16.15 (2.00) | | 14.23 (1.43) | 16.17 (2.00) | | 14.39 (1.49) |  |
| MGS, N/m, mean (SD) or median (IQR) | 248.17 (53.33) | 261.14 (30.79) | 288.83 (29.67) | | 249.07 (23.07) | 267.91 (23.58) | | 257.38 (22.71) |  |
| LGS, N/m, mean (SD) or median (IQR) | 295.60 (23.02) | 294.55 (25.64) | 314.17 (26.92) | | 269.07 (29.62) | 298.41 (27.32) | | 282.17 (21.00) |  |
| FMA-LE, mean (SD) | 18.29 (7.73) | 19.79 (7.47) | 20.29 (7.29) | | 22.36 (6.13) | 18.71 (4.75) | | 20.29 (4.67) |  |

Abbreviations: ESWT, extracorporeal shock wave therapy; MAS, modified Ashworth scale; IQR, interquartile range; PROM, passive range of motion; SD, standard deviation; MGF, medial gastrocnemius muscle oscillation frequency; LGF, lateral gastrocnemius muscle oscillation frequency; MGS, medial gastrocnemius muscle dynamic stiffness; LGS, lateral gastrocnemius muscle dynamic stiffness; FMA-LE, Fugl-Meyer assessment for lower extremity. T0, evaluation at baseline; T2, evaluation after three weeks treatment. Continuous variables are expressed as means (SD) or medians (IQR); while ordered categorical variables are described using medians (IQR).
